# Supplementary figures and images for: Modulation of fatty acid elongation in cockroaches sustains sexually dimorphic hydrocarbons and female attractiveness
Source: PLoS Biol. 2021 Jul 27;19(7):e3001330. doi: 10.1371/journal.pbio.3001330 (PMC8315507; doi:10.1371/journal.pbio.3001330)

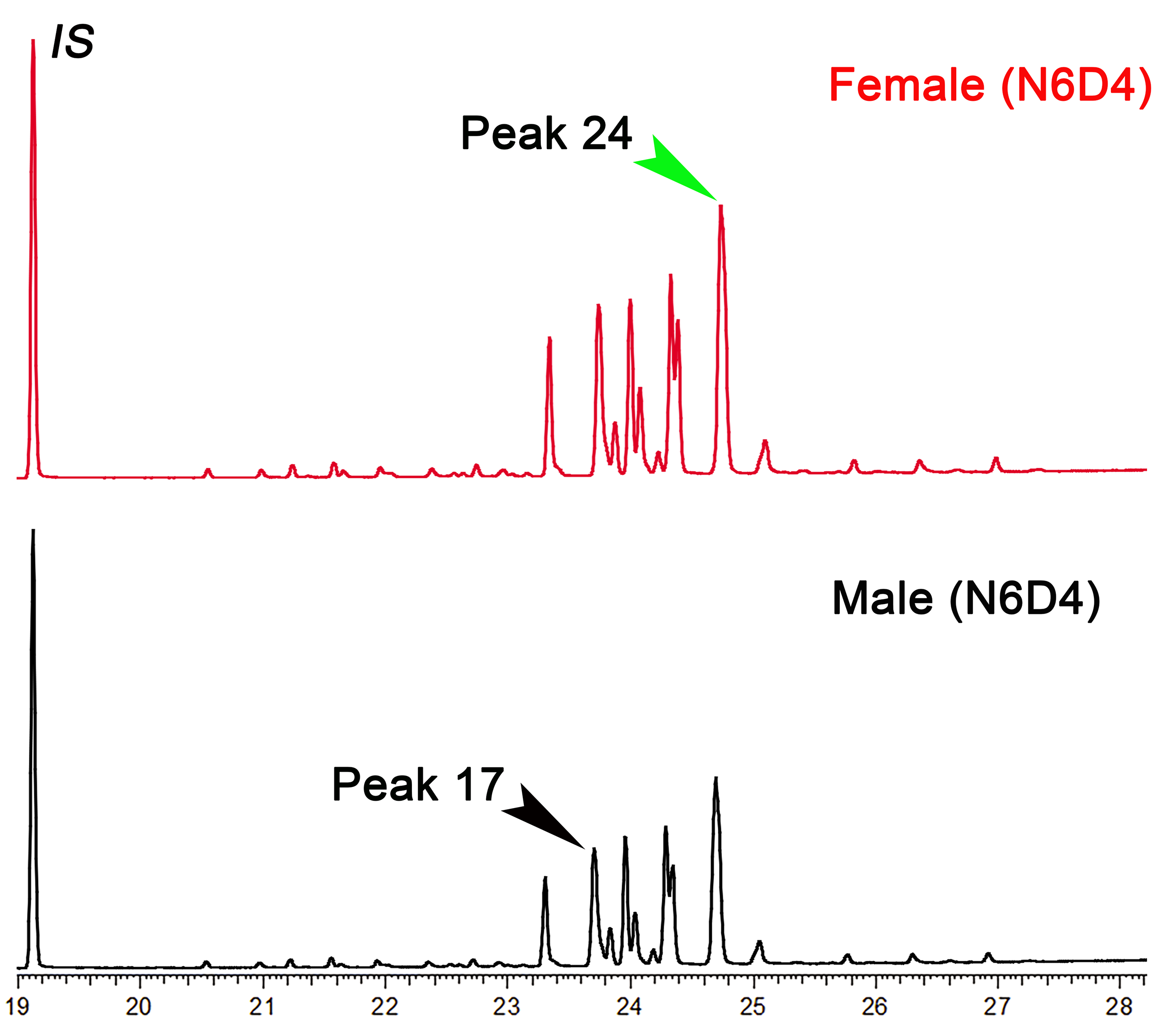

Supplement: S1 Fig — Peak 24 represents the female-enriched 3,7-; 3,9-; and 3,11-DimeC29, and Peak 17 is the male-enriched 9-; 11-; 13-; and 15-MeC29. The data underlying this figure are included in S2 Data. CHC, cuticular hydrocarbon. (TIF) [file pbio.3001330.s001.tif]

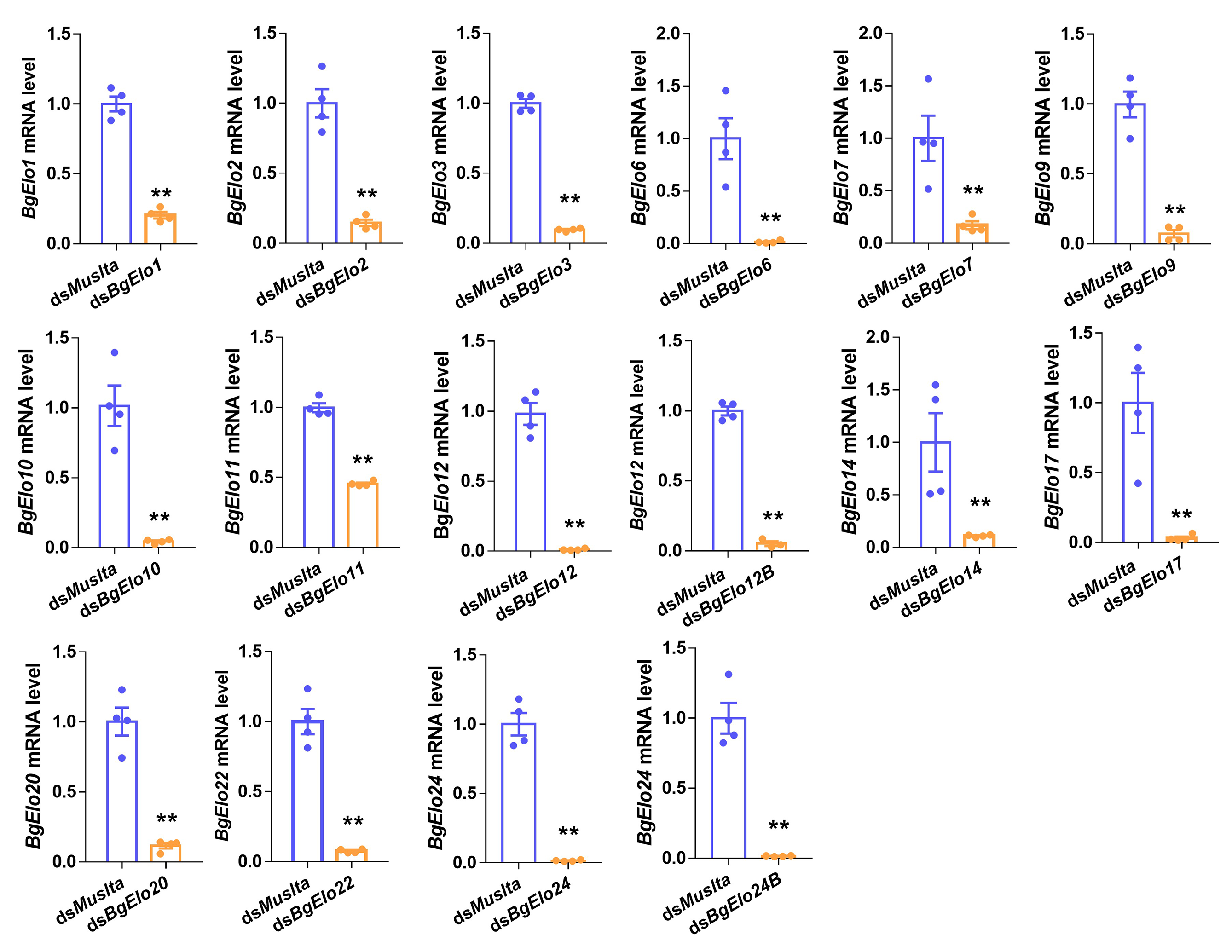

Supplement: S2 Fig — Data are shown as mean ± SEM, calculated from 3 to 4 replicates (2–3 cockroaches/replicate); **P < 0.01, 2-tailed Student t test. The data underlying this figure are included in S2 Data. RNAi, RNA interference. (TIF) [file pbio.3001330.s002.tif]

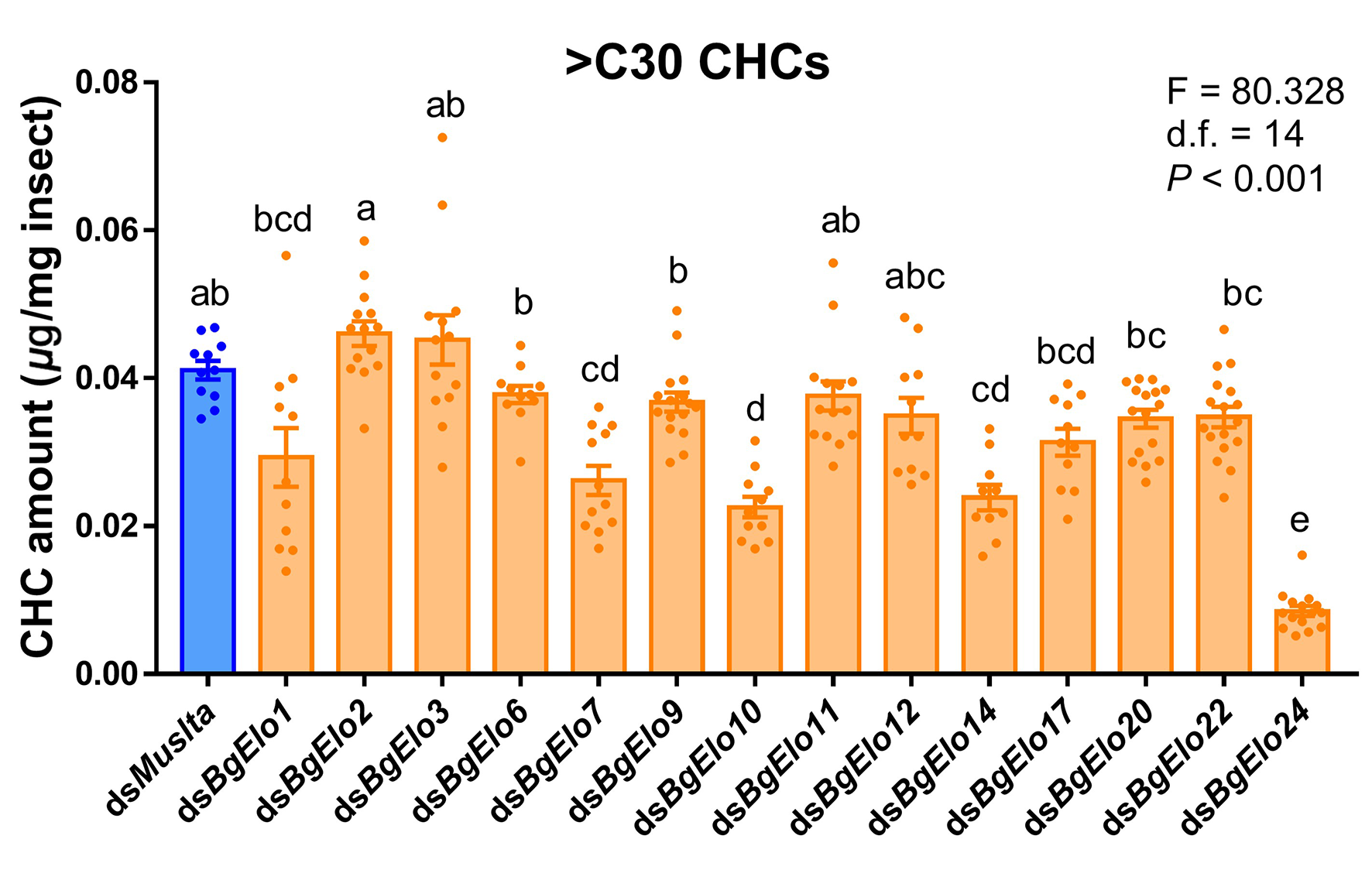

Supplement: S3 Fig — Different letters indicate significant differences between groups using Welch ANOVA (Games–Howell multiple comparisons test, P < 0.05). The data underlying this figure are included in S2 Data. CHC, cuticular hydrocarbon; RNAi, RNA interference. (TIF) [file pbio.3001330.s003.tif]

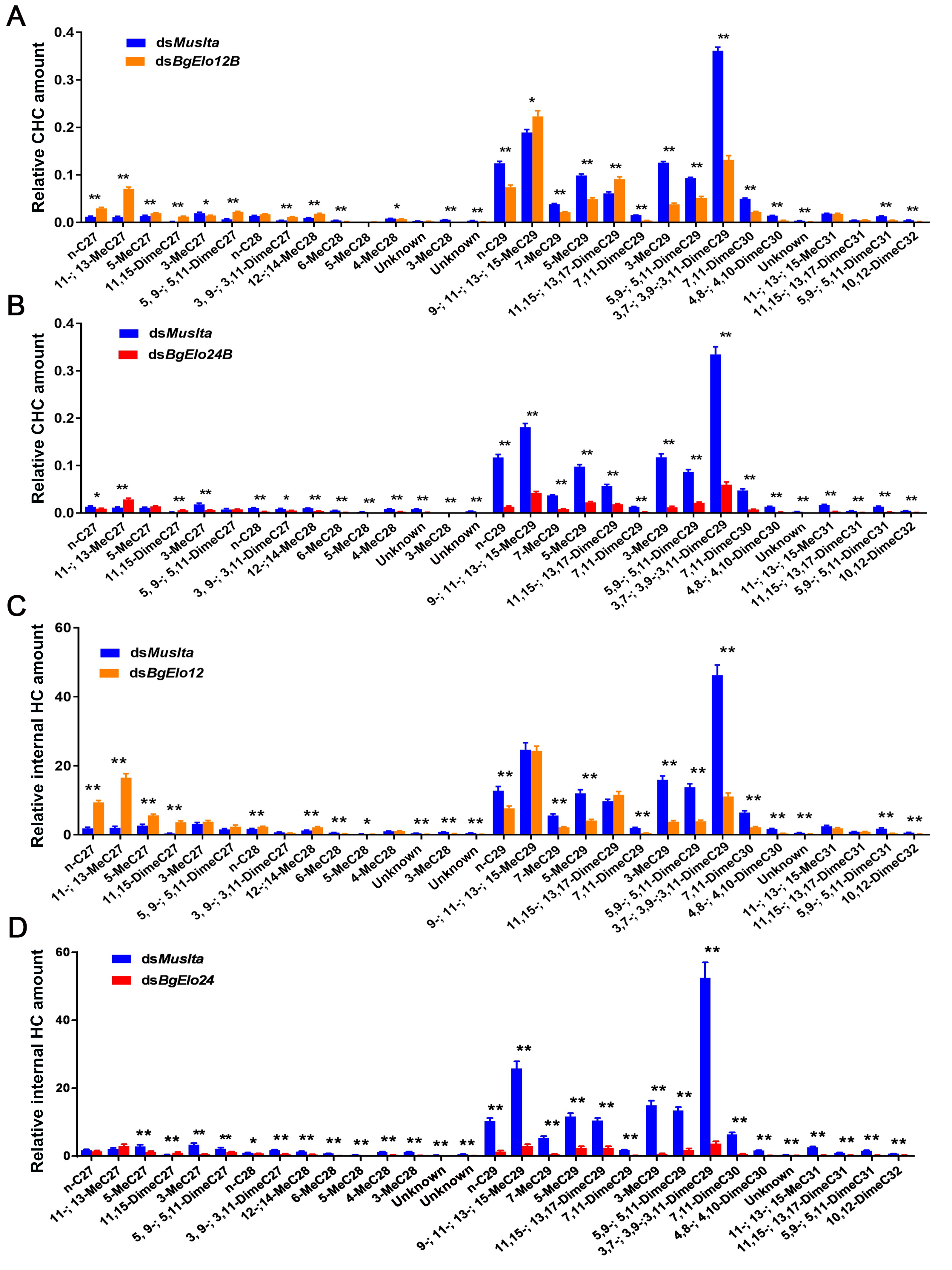

Supplement: S5 Fig — (A) Analysis of CHCs after RNAi of BgElo12 using the second target (dsBgElo12B) (B) or after RNAi of BgElo24 using the second target (dsBgElo24B). (C, D) The effects of BgElo12-RNAi and BgElo24-RNAi on internal HCs. Data are shown as mean ± SEM; *P < 0.05, **P < 0.01; 2-tailed Student t test, n = 9–12. The data underlying this figure are included in S2 Data. CHC, cuticular hydrocarbon; HC, hydrocarbon; RNAi, RNA interference. (TIF) [file pbio.3001330.s005.tif]

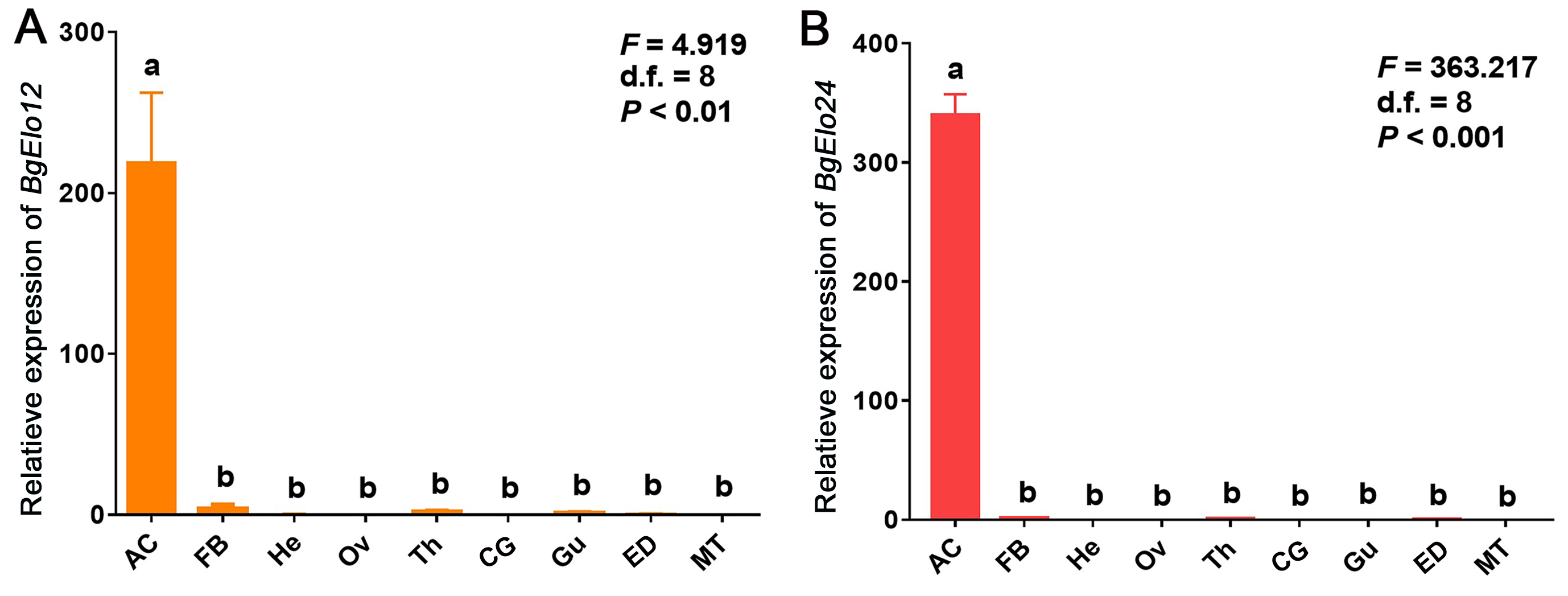

Supplement: S6 Fig — Data are shown as mean ± SEM; and each sample was collected from 4 (AC, Th, and Gu), 8 (FB, He, Ov, CG, and ED), and 12 (MT) cockroaches. Different letters indicate significant differences between groups using Welch ANOVA (Games–Howell multiple comparisons test, P < 0.05), n = 4. The data underlying this figure are included in S2 Data. AC, abdominal cuticle; Cg, colleterial gland; ED, ejaculatory duct; FB, fat body; Gu, gut; He, head; MT, Malpighian tubule; Ov, ovaries; Th, thorax. (TIF) [file pbio.3001330.s006.tif]

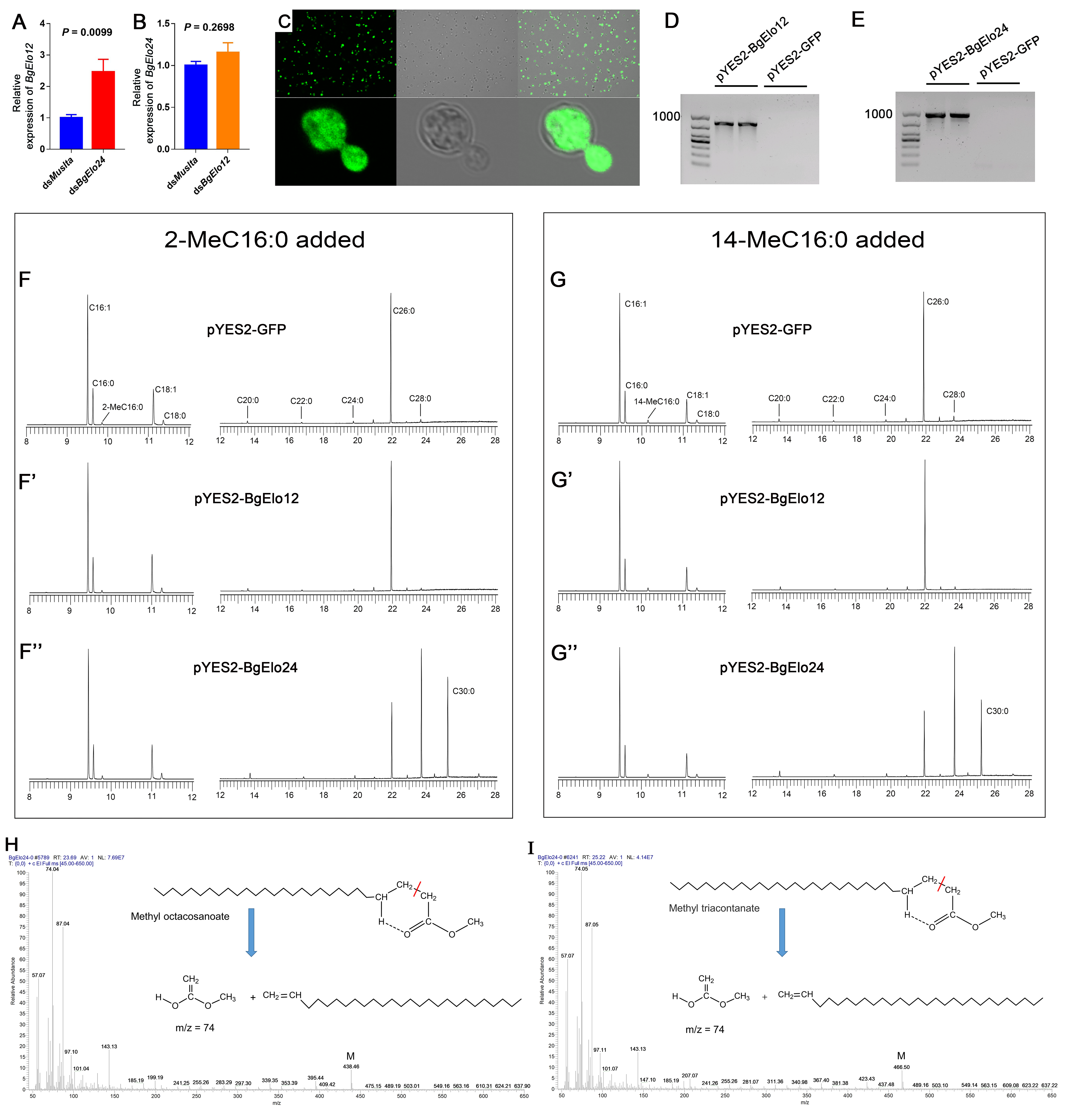

Supplement: S7 Fig — RNAi of BgElo24 up-regulated the expression of BgElo12 (A), while RNAi of BgElo12 did not affect BgElo24 transcript level (B). Data are shown as mean ± SEM; P values were calculated from 4 samples; each sample contained 2 cockroaches; 2-tailed Student t test. (C) Detection of GFP protein in the yeast with pYES2-GFP using a FV3000 confocal fluorescence microscope (Olympus). (D, E) RT-PCR analysis of the BgElo12 and BgElo24 mRNA after the induction with galactose. (F, F’, F”) Gas chromatograms of FAMEs after adding 2-MeC16:0 into the medium. (G, G’, G”) Gas chromatogram of FAMEs after adding 14-MeC16:0 into the medium. The compositions with retention times between 12 and 28 minutes were magnified about 50 times. (H, I) Mass spectra of methyl octacosanoate and methyl triacontanate, both of which showed a strong characteristic ion fragment (m/z = 74) and M peak. The data underlying S7A and S7B Fig are included in S2 Data. FAME, fatty acid methyl ester; RNAi, RNA interference; RT-PCR, real-time PCR. (TIF) [file pbio.3001330.s007.tif]

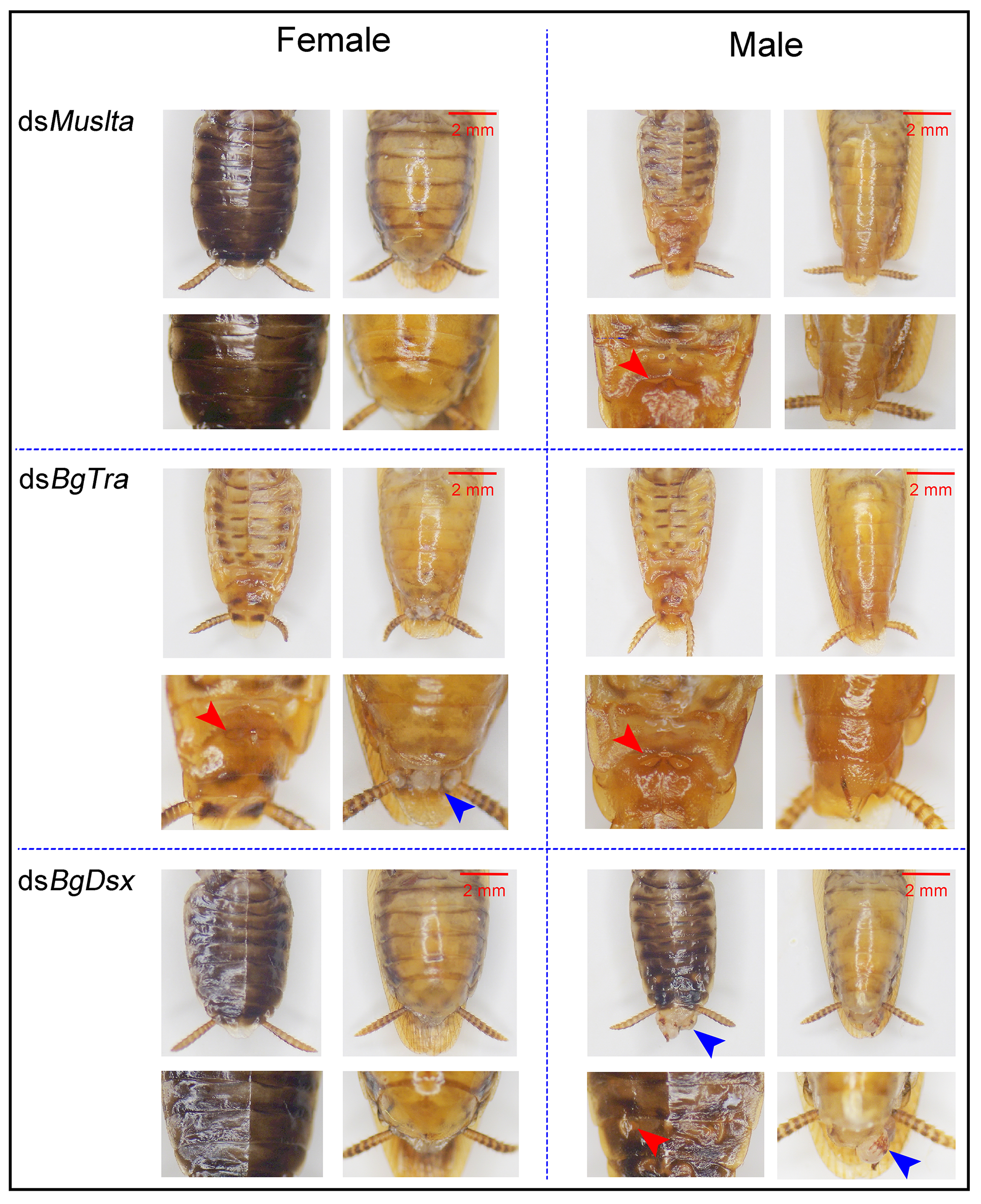

Supplement: S8 Fig — RNAi of BgTra in females generated a male-like body size and cuticle color, male-like tergal gland structure, and a protruding tissue at the end of the abdomen (left center); RNAi of BgDsx in males generated a female-like body color and a protruding tissue at the end of the abdomen, and the tergal gland partly disappeared (right bottom). Other treatments did not generate obvious developmental effects. RNAi, RNA interference. (TIF) [file pbio.3001330.s008.tif]

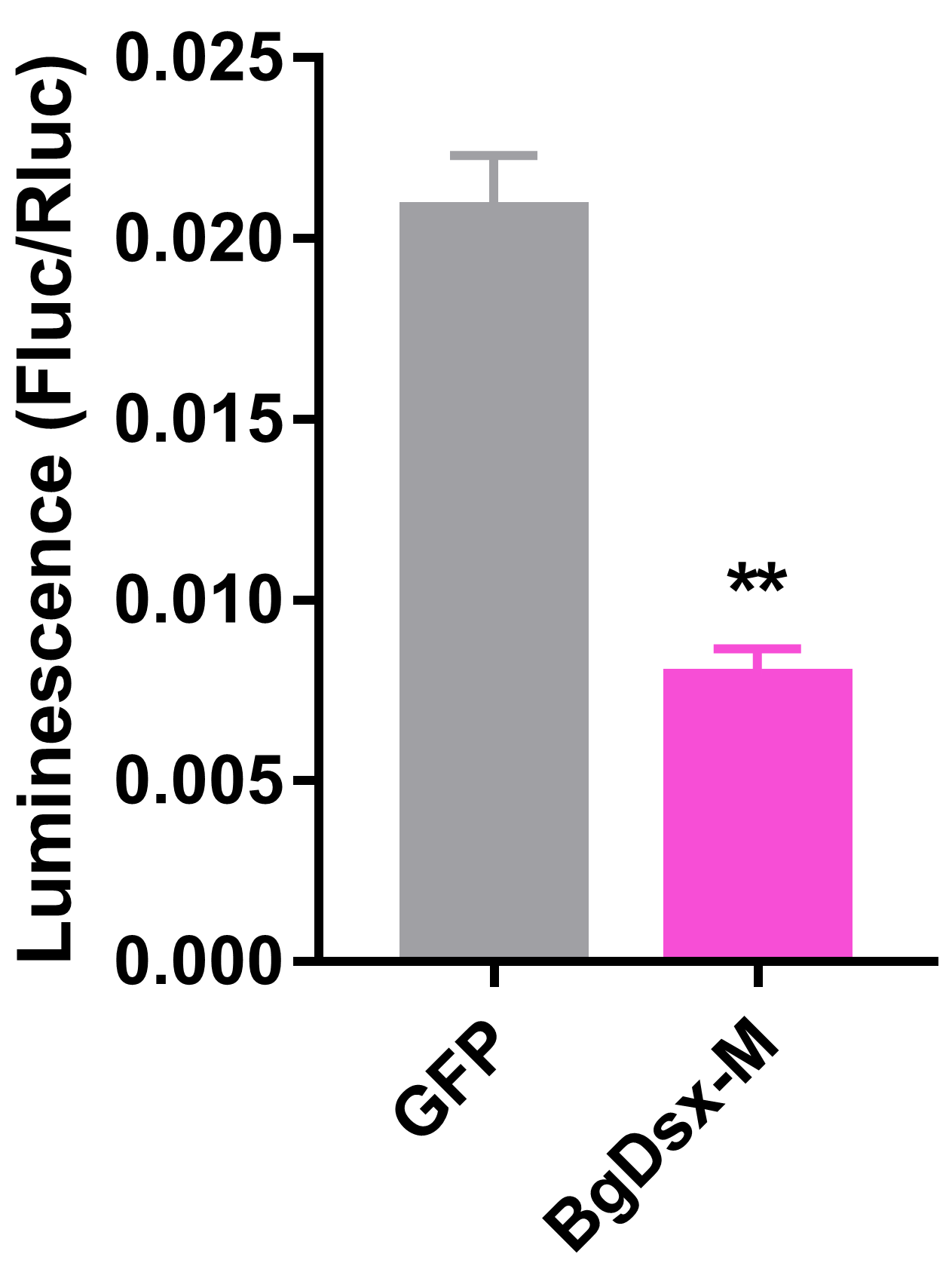

Supplement: S9 Fig — Data are shown as mean ± SEM; P values were calculated from 12 replicates; 2-tailed Student t test. The data underlying this figure are included in S2 Data. (TIF) [file pbio.3001330.s009.tif]

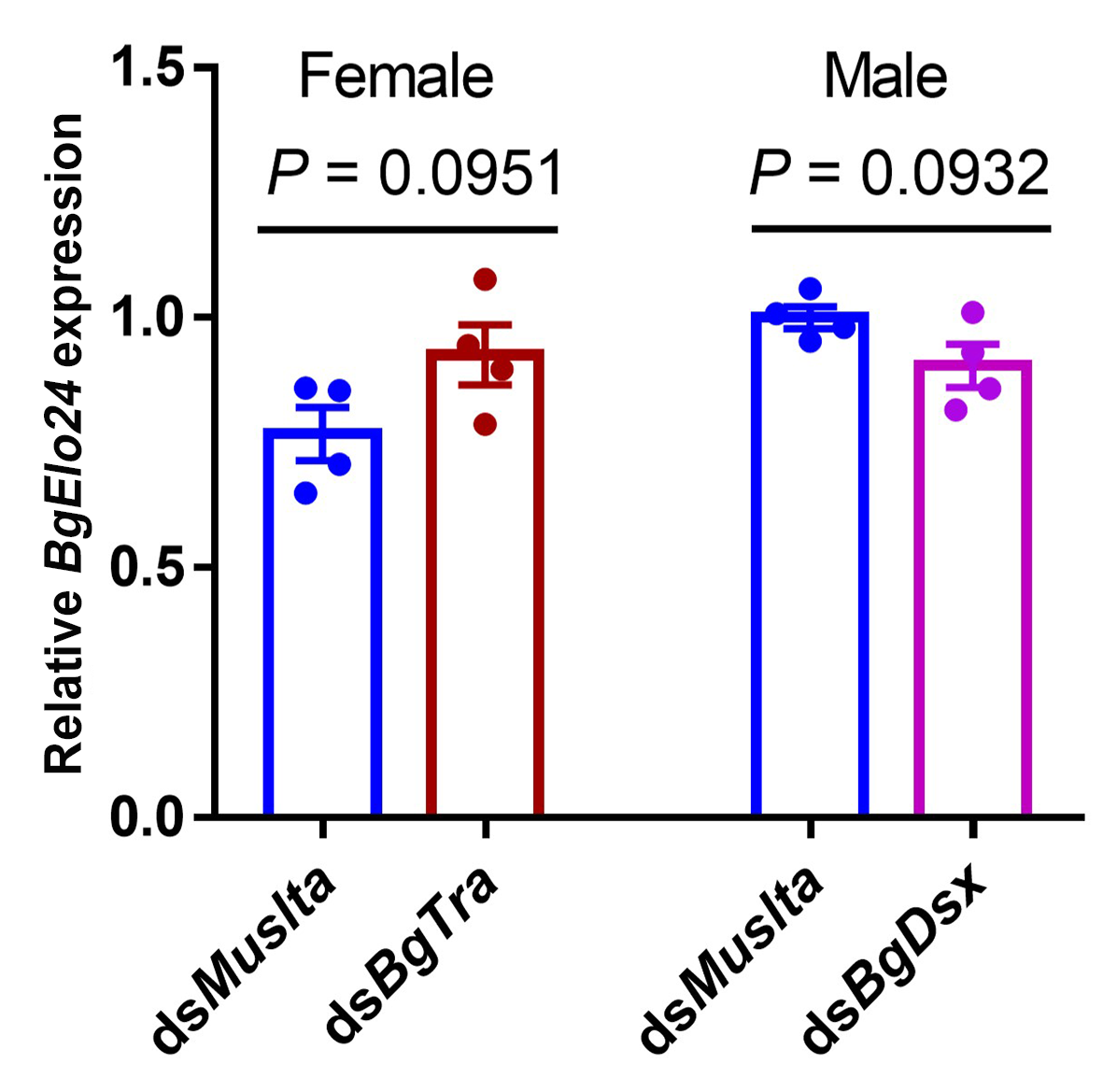

Supplement: S10 Fig — Data are shown as mean ± SEM; P values were calculated from 4 replicates (2 cockroaches/replicate); 2-tailed Student t test. The data underlying this figure are included in S2 Data. RNAi, RNA interference. (TIF) [file pbio.3001330.s010.tif]

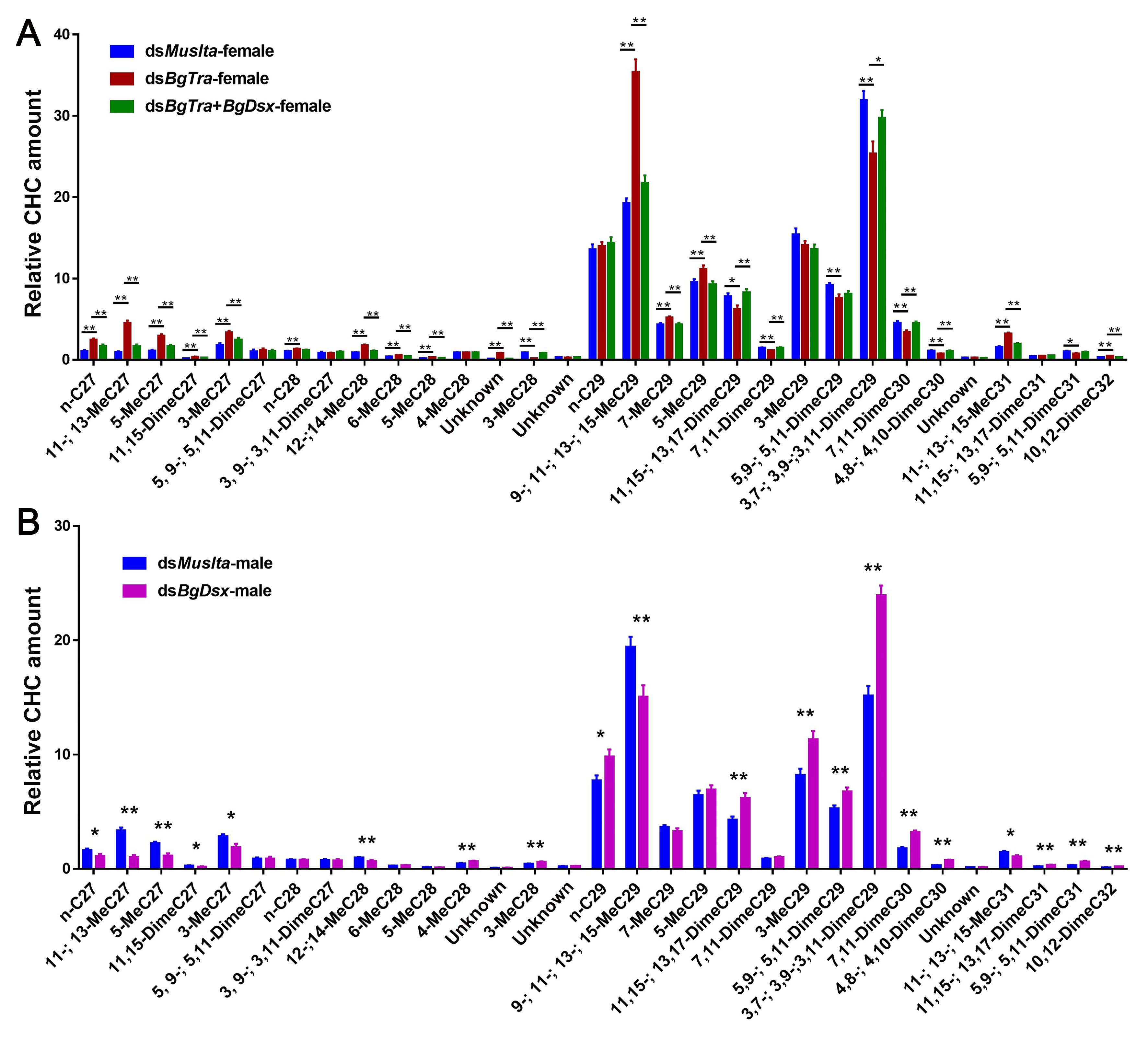

Supplement: S11 Fig — Data are shown as mean ± SEM, *P < 0.05, **P < 0.01, 2-tailed Student t test, n = 14 (dsMuslta-female), 12 (dsBgTra-female), 14 (dsBgTra+dsBgDsx-female), 12 (dsMuslta-male), and 16 (dsBgDsx-male). The data underlying this figure are included in S2 Data. (TIF) [file pbio.3001330.s011.tif]

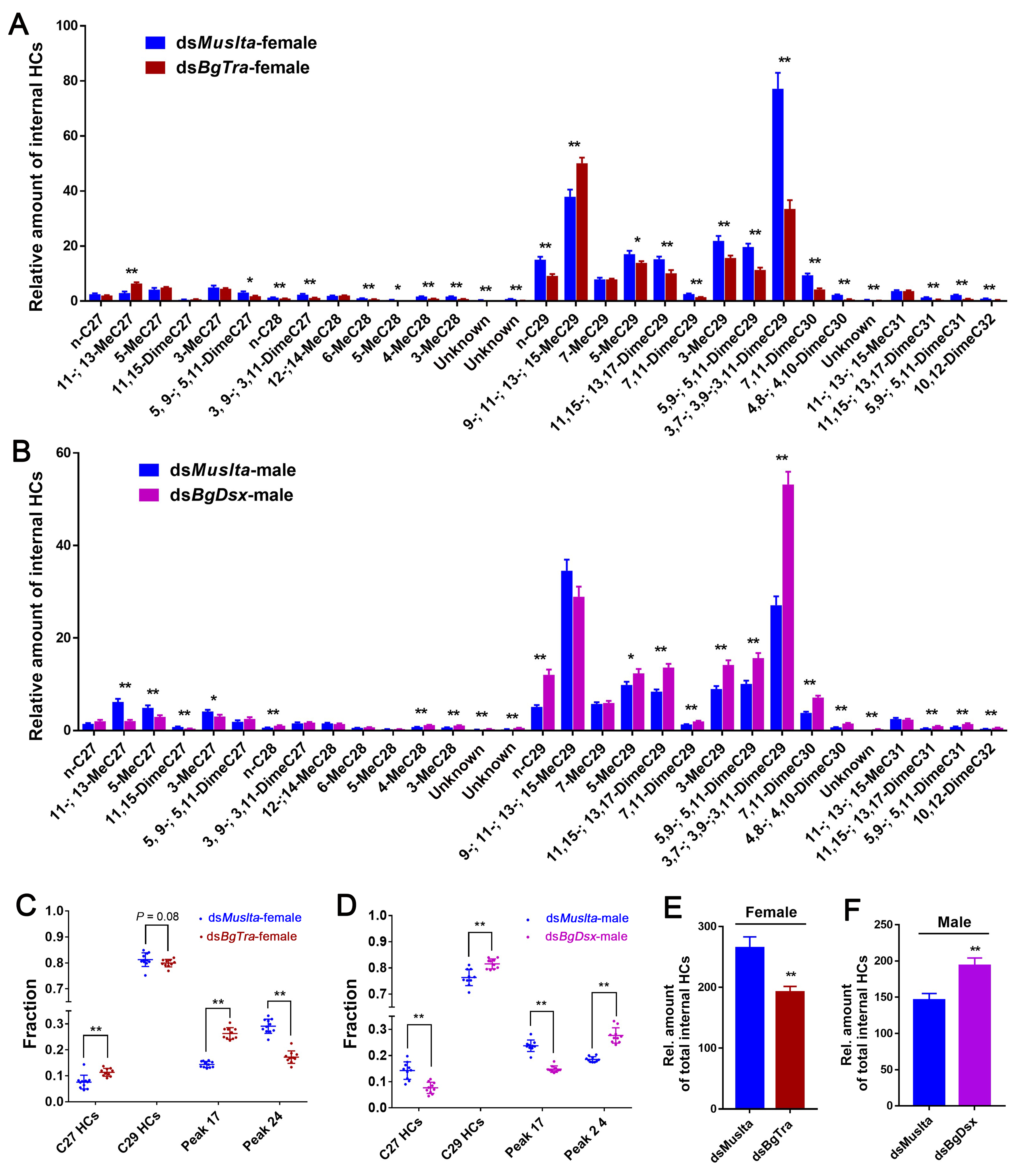

Supplement: S12 Fig — (A, B) Effects of BgTra-RNAi in females and BgDsx-RNAi in males on sex-specific internal HC profiles. (C, D) Proportion changes of representative internal HCs after RNAi of BgTra in females and BgDsx in males. (E, F) Effects of BgTra-RNAi in females and BgDsx-RNAi in males on total amounts of internal HCs. Data are shown as mean ± SEM, *P < 0.05, **P < 0.01, 2-tailed Student t test, n = 10 or 11. The data underlying this figure are included in S2 Data. HC, hydrocarbon; RNAi, RNA interference. (TIF) [file pbio.3001330.s012.tif]
