# Supplementary material for: Modulation of fatty acid elongation in cockroaches sustains sexually dimorphic hydrocarbons and female attractiveness
Source: PLoS Biol. 2021 Jul 27;19(7):e3001330. doi: 10.1371/journal.pbio.3001330 (PMC8315507; doi:10.1371/journal.pbio.3001330)
Supplement: Attachment — Submitted filename: Respons to reviewers.docx [file pbio.3001330.s020.docx]

Dear editor,

Thank you for your letter regarding the revision of our submitted manuscript entitled “Modulation of fatty acid elongation sustains sexually dimorphic hydrocarbons and female attractiveness in *Blattella germanica* (L.)” (PBIOLOGY-D-21-00844R1).

First, we would like to express our great appreciation to you and the four anonymous reviewers for the excellent comments, suggestions, and giving us a chance to revise our manuscript. We have carefully considered each of the comments and made point to point corrections. The responses to yours and reviewer’s comments are listed as below.

We do think that the revised version of this manuscript has been significantly improved. However, we would like to work with if further revision is needed.

We are looking forward to hear your decision soon.

Yours sincerely,

Tong-Xian Liu and Yong-Liang Fan

**Editor’s comments:**

**a)** Please change your title to "Modulation of fatty acid elongation in cockroaches sustains sexually dimorphic hydrocarbons and female attractiveness"

**Response:** Thanks for your suggestions, we have changed the title as suggested.

**b)** Please provide a blurb, according to the instructions in the submission form.

**Response:** We have provided a blurb file as required. Please refer to the attached file “Blurb”.

**c)** When revising your manuscript, please bear in mind the need to make the paper accessible to our broad readership (for example, we like reviewer #2’s suggestion that you depict the biosynthetic pathway in one of the Figures).

**Response:** We have simplified some of the statements in the article. Please refer to the revised file with mark-ups. Also, we have provided a hydrocarbon biosynthetic pathway in Fig 1H to make it easier to be understandable to broad readers.

**d)** Please attend to all of the requests from the reviewers.

**Response:** We have responded to the reviewers' comments one by one. Please see them at below.

**e)** Please attend to my Data Policy requests further down. Specifically, we note that you have provided some of the underlying data in the Supplementary Data files, but we think that data for some of the panels have not been presented separately. In addition, you should clearly cite the location of the data in each relevant Figure legend.

**Response:** Thanks. According to the requirements, we provided all the underlying data in supplementary data files. They were separately presented, and the correspondence was shown in relevant Figure legends and the titles of data files.

**Reviewer #1’s comments:**

**Comment 1:** Line 25. Correct the spelling of /underling'.

**Response:** We have corrected this mistake. See line 26.

**Comment 2:** Line 70. I think that it would be useful to describe in a little more detail that the elongase they are looking at is the first step and rate limiting step in elongation, which is then followed by they three steps indicated, reduction of the ketone to an alcohol, dehydration of the alcohol to the alkene and then reduction of the alkene to a saturated fatty acyl-CoA.

**Response:** This is a good comment. We have added more descriptions of the biochemical processes involved in the synthesis of very long-chain fatty acids. See line 70–73.

**Comment 3:** Line 422. ....only n-alkanes and... with the n italicized.

**Response:** Thanks. We have corrected it. See line 410.

**Comment 4:** Line 479. One of the best examples of hormonal regulation of chain length of hydrocarbons is the housefly, where ecdysone results in C27 alkenes being produced to where C23 alkenes become the dominant alkene.

**Response:** Thanks for your excellent comment. We have added this point. See line 475–479.

**Reviewer #2’s comments:**

**Main questions:**

Despite the overall excellence of the technical work, some of the data need to be reanalyzed with proper statistical tests and more details are needed about the methods. The significance of the findings needs to be more explicitly stated for a general science audience. The role of the sex determination pathway in sexually dimorphic morphological features is well-documented. Aside from Drosophila, are there other examples of the SD cascade in controlling CHCs or pheromones? This manuscript would be greatly improved with the editing of a native English speaker. In addition, if this article is meant for a broad audience, in its current form the text is rather dense. There are also some experiments and observations included that are somewhat tangential and should be omitted (details below)

**Response:** To our knowledge, regulation of CHCs or pheromones biosynthesis by sex-differentiation pathway has not been studied in other insects. This manuscript has been improved by native English speakers. And detailed responses for other suggestions are shown as below.

**Comment 1:** 61: Actually, the enzymatic pathways are well-characterized in multiple insect orders, as evidenced by the references provided in the rest of the paragraph and multiple reviews (Howard and Blomquist, 2005; Chung and Carroll, 2015; Blomquist and Bagneres, 2010; Martin and Drijfhout 2009; Yew and Chung 2015). Authors should consider including a pathway illustrating the biosynthesis of HCs to accompany this portion of the introduction.

**Response:** Thanks for your valuable suggestions. Combine your suggestions and those of the editors, we added a putative HC biosynthesis pathway in Fig 1H.

**Comment 2:** 74: replace "genes" with "enzymes". Again, I take issue with the statement that the genes/ enzymes are not well-studied. fact that the genes have been studied in few inspect species.
**Response:** Thanks. We have replaced "genes" with "enzymes". Yes, we agree that these enzymes or genes have been studied in few insect species, and here, we mainly want to emphasize that there are very few studies on their function in generation sexually dimorphic HCs (SDHCs).

**Comment 3:** 99-103: section on courtship can be shortened since this is discussed in detail in the results section.

**Response:** Thanks. We have accepted this suggestion. Please refer to lines 102–104.

**Comment 4:** 172: "RNAi of the genes" is lab jargon. I suggest replacing "RNAi" with "knockdown"
**Response:** We have accepted this suggestion.

**Comment 5:** 220: N6D4: Please define in the text. It is only explained in Fig 6 legend.
**Response:** We have added an annotation for N6D4 at line 229.

**Comment 6:** 228-229: awkwardly worded - "contributes to" in place of "support"? Also, the statement that "the role of Elo24 is desication tolerance" is overly general, based on a single assay. The enzyme may contribute to desiccation tolerance but it likely has other functions.
**Response:** We’ve reorganized this sentence**.** Please refer to lines 236–238.

**Comment 7:** 246: It looks like HCs in the C27 - C32 range were downregulated, not just C28-29.

**Response:** Yes, we have noticed most of the HCs were downregulated, but 11,13-DimeC27 and 11,15-DimeC27 were upregulated, so we didn't mention C27 HCs, and we have changed “C28–C29” to “C28–C32”. Please refer to line 257.

**Comment 8:** 255-256: Sentence could be reworded to make it more explicit what GFP expression indicates.
**Response:** We have reworded this sentence. Please refer to lines 267–268.

**Comment 9:** 308 - 317: This section could be shortened and the explanation of age used for assays can go in methods
**Response:** We have shortened this section, and moved the explanation to methods. Please refer to lines 652–655.

**Comment 10:** 370-383: The observations about internal vs cuticular HC levels in this section are interesting but confusing in light of results showing that BgElo12 RNAi does not impact HC transport. The findings are also tangential to the main results and the underlying mechanisms can only be speculated upon at this point. I suggest drastically shortening this section, moving to the discussion section, or removing.

**Response:** Thanks for your suggestions. We did not remove this section entirely, but we have shortened this section and moved a few sentences to the Discussion. We think this section may be useful in explaining that the function of sex-differentiation genes in sexually dimorphic HCs is largely relay on the regulation of *BgElo12* expression, but not selective transport of HCs from internal tissues to cuticle. Please refer to lines 441–450.

**Comment 11:** 453-455: Sentence construction is awkward.

**Response:** We have reorganized this sentence. Please refer to lines 452–454.

**Comment 12:** 478-479: The 20-HE steroid dehydrogenase was also identified as a regulator of HC profiles:

Chiang YN, Tan KJ, Chung H, Lavrynenko O, Shevchenko A, Yew JY. Steroid Hormone Signaling Is Essential for Pheromone Production and Oenocyte Survival. PLoS Genet. 2016 Jun 22;12(6):e1006126. doi: 10.1371/journal.pgen.1006126. PMID: 27333054; PMCID: PMC4917198.
**Response:** Thanks for your excellent comment, and we have added this point and referred it in our manuscript. Please refer to line 479.

**Comment 13:** 488-489: Sentence construction is awkward.
**Response:** We have reorganized this sentence. Please refer to lines 488–490.

**Comment 14:** 494-497: I suggest removing text related to krh1. If authors think this is an important result, it should go into results section. However, the data provided are not sufficient to support the conclusion in 498-499.
**Response:** Thanks. We accepted your suggestion and removed this Krh1 section.

**Comments on method:**

**Comment 1:** How were the identifies of chromatogram peaks identified?

**Response:** In fact, HC components from *B. germanica* have been well studied, identification of HC peaks was performed as described previously：Jurenka, R. A., Schal, C., Burns, E., Chase, J., Blomquist, G. J. (1989). Structural correlation between cuticular hydrocarbons and female contact sex pheromone of German cockroach *Blattella germanica* (L.). Journal of chemical ecology, 15, 939–949. We have added this reference in the Methods.

**Comment 2:** How was quantitation of GCMS peaks performed? The numbers provided in S1 and S2 tables are presented as absolute quantities. However, absolute quantification would require comparing the intensity of the peaks to a synthetic version of the compound. It does not appear that synthetic standards were used. The tables and figures should indicate that "relative quantities" are measured rather than absolute amounts. More details are also needed as to how percentage (e.g., 1B-E; 3D, E) was calculated. Is it percentage of all peaks detected or just percentage of HCs?

**Response:** In this study, quantification of different HCs was performed through a commonly used internal standard method (Weng et al. 2013. miR-124 controls male reproductive success in *Drosophila*. eLife, 2, e00640; Bilen et al. 2013. Regulation of onset of female mating and sex pheromone production by juvenile hormone in *Drosophila melanogaster*. PNAS, 110, 18321–18326.). When preparing the hydrocarbon samples, 15 µg of *n*-Hexacosane were added into the sample as an internal standard. See line 640–641. In order to avoid controversy, we’ve replaced absolute content with relative content. See S1 and S2 table.

Using this HC extraction method, almost all detected GC peaks are hydrocarbons as indicated by the characteristic ion (m/z = 15+14n) and previously identified by Jurenka et al. 1989. We calculate the proportions of different HC components in the total HCs.

**Comment 3:** Internal HC extractions: the fat body is diffuse throughout the body of the insect. How was it dissected in a standardized way from individual to individual? The reference (Fan et al., JEB 2008) did not provide more details.

**Response:** In fact, internal HCs not only include the HCs in fat bodies; the ovary, the hemolymph, and other tissues or organs are also the major HCs storage sinks (Schal et al. 1998. Sites of synthesis and transport pathways of insect hydrocarbons: cuticle and ovary as target tissues. Integr Comp Biol, 38: 382–393.). Therefore, we firstly removed the cuticular HCs from cockroaches, and the whole cockroach without cuticular HCs was further homogenized and used for the internal HCs extraction. Please refer to lines 537–541.

**Comments on Figures or Tables**

**Comment 1:** S1, S2 table: "ug" in the footnote should be replaced with "μg". Please add sample size to footnote. If these numbers were calculated based on the relative intensity of the compound to the internal standard, they are relative quantities rather than absolute quantities. Table title should be "Relative quantification…"

**Response: Thanks.** We have accepted all your suggestions. See S1, S2 table.

**Comment 2:** S4, 5: See comment for Fig 3.

**Response:** Thanks for your suggestions, we have explained it in Response to Comment 4.

**Comment 3:** S6 7A: What is the unit of y-axis?

**Response:** That's the relative expression level of target gene, we have changed the presentation and added “Relative”. See S6, S7A, and S7B Fig.

**Comment 4:** Fig 3B, C: An ANOVA is needed here to compare the relative amounts of individual CHCs rather than multiple t-tests. Before ANOVA can be performed, the CHCs should be collapsed into fewer categories so that the number of categories is less than the sample size. Y-axis should indicate that these are relative quantities.

**Response:** Thanks for your suggestions. We have considered these suggestions carefully. However, we think it is necessary to show the effect of inhibition of *BgElo12* or *BgElo24* on individual hydrocarbon components, as there are some selective effects on individual HCs, especially for *BgElo12*-RNAi. In addition, their influences on different chain-length CHCs (a fewer categories manner) have been shown in Fig 2.

For the statistical method, we considered that there was only one treatment group (dsBgEloX) and one control group (dsMuslta), our purpose was to compare the difference of the same HC component between control and treatment. So we think *t*-test may be more appropriate here. However, we can keep discussing about it.

We have accepted your suggestion and changed Y-axis. See Fig 3B, 3C, S4, and S5 Fig.

**Comment 5:** Fig 3D: comparison of each CHC type between 3 different groups requires an ANOVA rather than multiple *t*-tests.

**Response:** Thanks. We have accepted your suggestions. See Fig 3D.

**Comment 6:** Fig 5A: Was the C29 methyl ketone quantified using a synthetic standard or do numbers represent abundance relative to internal standard?

**Response:** It was relative abundance, we have changed it in Fig 5A, 5C, and 5D.

**Comment 7:** Fig 6F: ANOVA should be used to compare between the 3 genotypes.

**Response:** Thanks. We have accepted your suggestions. See Fig 6F.

**Reviewer #3’s comments:**

**Comment 1:** In the RNAi efficiency experiments, it is not clear which tissue was used to performed these experiments.

**Response:** In RNAi efficiency analysis, intact cockroaches were used for total RNA extraction and RT-qPCR. We have added this information in the methods. See line 606.

**Comment 2:** Lines 185-186: the authors wrote "we cannot rule out that other BgElo genes might have a less prominent role in HC biosynthesis". I think it is necessary to specify that this statement applies to the very long chain hydrocarbons (C27-C30), to reflect the data presented in the paper.
**Response:** Thanks for your suggestion, we have changed it. See line 193–194.

**Comment 3:** Figure 2: while there is nothing wrong with using the Games-Howell multiple comparisons test, I think it would be more appropriate to use Dunnet's procedure and test dsBgEloX treatment vs dsMuslta control. The conclusions would most certainly not be affected.
**Response:** Thanks. We agree with your opinion that Dunnett’ *t* test is more suitable at this condition to compare the differences between ds*BgEloX* and ds*Muslta*. However, test of homogeneity of variances was failed here, therefore a less than perfect multiple comparisons test was chosen based on an unequal variances assumption.

**Comment 4:** Figure 3: It looks like knocking down Elo24 is leading to less CHCs overall. It might be useful to visualize the data using percentages (absolute quantities). Also, I think it may be helpful to provide a PCA with the different treatments. Similarly to what authors do in Fig 1, a PCA plot would help the readers see the similarities and differences between female and male profiles and the impact of the treatments.

**Response:** Thanks for your comments. The percentage changes of the most representative CHC components (As indicated by Wexler et al., 2019, eLife) were shown in Fig 3D. In addition, according to your suggestions, we’ve performed a PCA using the percentages of different CHC components. Results are shown in S4C and S4D Fig, knockdown of *BgElo12* in females generated a similar distribution pattern with ds*Muslta*-male, but there was no overlap. We think this is mainly caused by the excessive repression of *BgElo12* (> 90%) by RNAi in our experiments. Naturally, *BgElo12* expression levels are only ~40 percent higher in females than in males, injection of ds*BgElo12* in females caused an excessive increase of male-enriched CHC components and an excessive decrease of female-enriched CHC components (Fig 3B). Moreover, the distribution of HC profiles from *BgElo24*-RNAi females is indeed far from both the ds*Muslta*-RNAi female and ds*Muslta*-RNAi male patterns.

**Reviewer #4’s comments:**

**Main question 1:** - Can the authors explain and rectify why they did not analyze the effects of the elongases on the fatty acid profiles but only the resulting hydrocarbon profiles?

**Response:** Thanks for your suggestions. Analysis of fatty acid profiles is useful for studying the function of BgElos. However, only C10–C20 fatty acid (no methyl branched fatty acid) were detectable in *Blattella germanica* (Pei et al, 2019. *BgFas1*: A fatty acid synthase gene required for both hydrocarbon and cuticular fatty acid biosynthesis in the German cockroach *Blattella germanica* (L.). Insect Biochem Mol Biol. 2019 Sep, 112:103203.). The amount of very long chain fatty acids and methyl branched fatty acids was too low to be detected, or they were quickly converted into HCs. CHCs from *B. germanica* were mainly composed of methyl branched long-chain (C27–C32) CHCs, therefore, we used yeast expression to study *BgElo12* and *BgElo24* functions.

**Main question 2:** - This appears especially important as they could not demonstrate the activity of BgElo12 and BgElo24 on the elongation of the methyl-branched fatty acids that have to be involved in the pheromone biosynthesis (cf. Lines 280-281).

**Response:** Yes, it is a pity that we can not demonstrate the activity of *BgElo12* and *BgElo24* in elongation methyl-branched fatty acids at the present study. We have tried many times and all were failed. We think this is mainly caused by the yeast expression system itself. To our knowledge, the catalytic elongation of methyl-branched fatty acids has never been studied in yeast, and we also found there was no methyl-branched fatty acid in *Saccharomyces cerevisiae*. The elongation of the fatty acids requires four enzymes (elongase, 3-keto-acyl-CoA-reductase, 3-hydroxy-acyl-CoA dehydratase, and *trans*-enoyl-CoA-reductase), and we only express the key rate-limiting enzymes, the three other enzymes that derived from the yeast itself may not recognize the methyl-branched substrates.

**Comment 1:** Line 25: The sentence starting "Here, we report…." Does not read well to me. English is not my native language but I wonder if "underling" is really the right word? I would suggest "underpins".

**Response:** We have reorganized this sentence and corrected the spelling according to your and reviewer 1’ comments. See line 25–26.

**Comment 2:** Line 28: I would suggest that the sentence is slightly rephrased: "RNAi screen against the fatty acid elongase family members combined with heterologous expression of the genes in yeast revealed that both…."

**Response:** Thanks for your suggestions, and we have changed it. See line 28–29.

**Comment 3:** Line 412: Delete "were"?

**Response:** We have corrected it. See line 402.

**Comment 4:** Line 505-506: While this is obviously true for moths (Lepidoptera) I wonder if it is generally true? Can the authors provide a reference?

**Response:** We have changed this overly strong statement, and we’ve replaced “insect” with moth. See line 498.

**Comment 5:** Fig 1: Black=Male and Red=Female seems to apply to all panels but G. I would suggest the authors consider changing the colour code in G and possibly move the Black/Red -Male/Female explanation out of the "Frame A" as it applies to all of the other panels.

**Response:** Thanks for your suggestions, we have changed it. See Fig 1.

**Comment 6:** Fig 2: What is the unit on in A and B?

**Response:** We have changed the y-axis, and it’s relative expression level of BgElos. See Fig 2A and 2B.

**Comment 7:** Fig 3: How do I find out what the different peak numbers stand for in A, B, C, D and E?

**Response:** Sorry for that, it’s our mistakes; they were indicated in S2_Data, and we have added this information in Fig 3 legends. See line 243–244.
